# Effects of Particulate Matter on Inflammation and Thrombosis: Past Evidence for Future Prevention

**DOI:** 10.3390/ijerph19148771

**Published:** 2022-07-19

**Authors:** Sasinee Hantrakool, Sirinart Kumfu, Siriporn C. Chattipakorn, Nipon Chattipakorn

**Affiliations:** 1 Department of Internal Medicine, Faculty of Medicine, Chiang Mai University, Chiang Mai 50200, Thailand; sasinee.h@cmu.ac.th; 2 Cardiac Electrophysiology Research and Training Center, Faculty of Medicine, Chiang Mai University, Chiang Mai 50200, Thailand; 3 Center of Excellence in Cardiac Electrophysiology Research, Chiang Mai University, Chiang Mai 50200, Thailand; siriporn.c@cmu.ac.th; 4 Cardiac Electrophysiology Unit, Department of Physiology, Faculty of Medicine, Chiang Mai University, Chiang Mai 50200, Thailand; 5 Department of Oral Biology and Diagnostic Sciences, Faculty of Dentistry, Chiang Mai University, Chiang Mai 50200, Thailand

**Keywords:** particulate matter, inflammation, oxidative stress, adhesion molecule, coagulation, thrombosis

## Abstract

Ambient air pollution has become a common problem worldwide. Exposure to pollutant particles causes many health conditions, having a particular impact on pulmonary and cardiovascular disease. Increased understanding of the pathological processes related to these conditions may facilitate the prevention of the adverse impact of air pollution on our physical health. Evidence from in vitro, in vivo, and clinical studies has consistently shown that exposure to particulate matter could induce the inflammatory responses such as IL-6, TNF-α, IL-1β, as well as enhancing the oxidative stress. These result in vascular injury, adhesion molecule release, platelet activation, and thrombin generation, ultimately leading to a prothrombotic state. In this review, evidence on the effects of particulate matter on inflammation, oxidative stress, adhesion molecules, and coagulation pathways in enhancing the risk of thrombosis is comprehensively summarized and discussed. The currently available outcomes of interventional studies at a cellular level and clinical reports are also presented and discussed.

## 1. Introduction

Air pollution has recently become a major concern worldwide. It has been proposed that exposure to high ambient pollutant particles leads to adverse health impacts which may contribute to as many as three million premature deaths per year [1,2]. There is extensive evidence to confirm the various adverse outcomes of the small particles on our health after both acute and long-term exposure [3]. These fine particles enter the blood circulation through the respiratory tract and quickly activate the pulmonary and systemic inflammatory responses. Activation of the inflammatory cytokines, oxidative stress, and adhesion molecules results in atherosclerosis [4,5,6]. Previous reports revealed a significant association between high ambient particulate matter and an increased incidence of cardiovascular disease including ischemic stroke [7,8], acute coronary syndrome [9,10,11], and thrombosis [12,13,14].

Particulate matter (PM) can consist of a variety of components depending on the source [15,16]. PM is defined according to particle size. Coarse particulate matter, PM_10_ has an aerodynamic diameter of between 2.5 to 10 microns. Fine particulate matter, PM_2.5_ has a particle size of more than 0.1 micron, but less than 2.5 microns in aerodynamic diameter. Ultrafine particulate matter (UFP) has an aerodynamic diameter of less than 0.1 micron [17,18]. Diesel exhaust particles (DEP) are the particles released from the combustion of fuel in diesel-fuelled vehicles and consist of a mixture of polycyclic aromatic hydrocarbon, organic compounds, sulfate, nitrate, other trace elements, and metals such as copper, iron, nickel, vanadium, and zinc [19]. These particles enter the body via inhalation. The coarse particles are mostly trapped in the upper airway, whereas smaller particles can pass beyond the lower airway, passing into the blood, causing adverse effects throughout the body [17,18]. Past evidence has demonstrated that PM could induce oxidative stress [20,21,22,23,24,25,26,27], result in DNA damage [25,28,29], and activate local and systemic inflammatory response [22,23,25,26,30]. Furthermore, PM has also been shown to impair vascular function [30,31], increase the expression of vascular inflammatory biomarkers, including intercellular adhesion molecules (ICAM-1), vascular cell adhesion molecules (VCAM-1), and P-selectin [24,32]. VCAM-1 and ICAM-1 and p-selectin are vascular adhesion molecules that play an important role in thrombus formation by promoting leukocyte-endothelial and leukocyte-platelet interaction during the inflammatory response [24,32]. Increased levels of these adhesion molecules instigate the recruitment of inflammatory cells into vascular endothelium resulting in the release of microparticles and the activation of platelet adhesion and platelet aggregation. Blood microparticles are small parts of cell membrane, secreted from various cell types, including endothelial cells, monocytes, and platelets, and containing both phosphatidylserine and tissue factor, the potent procoagulants, which further activate the down-stream coagulation cascade [33,34].

## 2. Hemostasis and the Fibrinolytic Pathway

**Figure 1:**
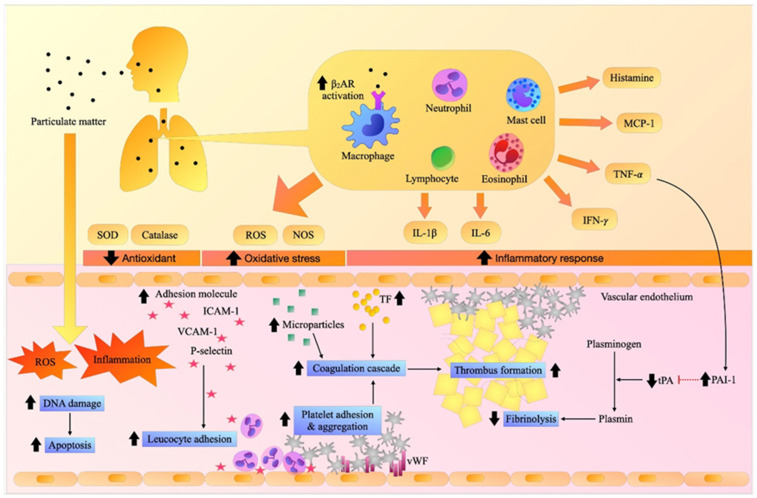
Effects of particulate matter (PM) on inflammation, oxidative stress, adhesion molecules, and thrombosis.

Normal hemostasis is a complex system mainly maintaining stable physiology in the body and protecting against pathological processes. The generation of thrombin is the key mechanism that links blood clot formation and the fibrinolytic system, the counterbalance that controls the thrombotic process [33,35]. In general, vascular endothelial injury induces platelet adhesion, platelet activation, and the release of the von Willebrand factor (vWF), tissue factor (TF), cytokines and chemokines. Primary hemostasis occurs when platelets adhere to the injured site, activating platelet aggregation, which is promoted by the vWF. TF is the main coagulation factor that initiates blood coagulation by activation of Factor VII in the extrinsic pathway, resulting in the generation of thrombin. This would further stimulate elements of the intrinsic pathway including Factors IX and VIII and then the downstream common coagulation pathway including Factors X and V, resulting in production of the prothrombinase complex (FVa-Xa), which generates excessive thrombin, leading to fibrin clot formation [33,35]. In the fibrinolytic pathway, tissue plasminogen activator (tPA) and plasminogen-activator inhibitor-1 (PAI-1) are the main regulators that modulate fibrinolysis. tPA activates and causes the cleavage of plasminogen into plasmin, resulting in the degradation of fibrin clots. PAI-1 is a serine protease inhibitor, which inhibits tPA, preventing clot lysis. An imbalance of tPA and PAI-1 levels therefore has an impact on fibrin clot formation [35]. Hemostasis and fibrinolytic pathways are summarized in Figure 1.

Inhalation exposure to PM induces pulmonary and systemic inflammation and oxidative stress. It increases the expression of adhesion molecules resulting in the recruitment of inflammatory cells and the activation of the vascular endothelium, platelets, and coagulation cascade, causing fibrin clots, while hampering fibrinolytic activity, and eventually contributing to thrombosis. β_2_AR: beta-2 adrenergic receptor, DNA: deoxyribonucleic acid, ICAM-1: intercellular adhesion molecules-1, IFN-γ. interferon- γ, IL-1β: interleukin-1β, IL-6: interleukin-6, MCP-1: monocyte chemoattractant protein-1, NOS: nitric oxide synthase, PAI-1: plasminogen activator inhibitor-1, ROS: reactive oxygen species, SOD: superoxide dismutase, TF: tissue factor, TNF-α: tumor necrosis factor-α, tPA: tissue plasminogen activator, VCAM-1: vascular adhesion molecule-1.

Currently, knowledge surrounding the outcomes of PM-mediated coagulation and fibrinolysis is still inconclusive. In this review, the effects of PM on the inflammatory responses, oxidative stress, adhesion molecules, and coagulation factors related to thrombosis from in vitro, in vivo, and clinical studies are comprehensively summarized and discussed. The understanding accrued from this overview of the pathological process of PM-mediated thrombosis will help in limiting or preventing the damaging effects of PM exposure on our health.

## 3. The Effects of Particulate Matter on Inflammation, Oxidative Stress, and the Coagulation System: Reports from In Vitro Studies

Over the past decade, it has been shown that polluted air particles can activate inflammation, and oxidative stress and cause cell death [36,37]. In vitro reports indicate that exposure to PM could enhance the inflammatory response and oxidative stress, activating the coagulation cascade and inducing cell death, leading to a prothrombotic state [38]. Vanadium pentoxide (V_2_O_5_) is one of the toxic substances, a consequence of burning fuel oil and fly ash. An in vitro study showed that V_2_O_5_ could directly affect the human umbilical vein endothelial cells (HUVECs), by enhancing oxidative stress, and increasing expression of adhesion molecules, which resulted in shape changes, decreased cell proliferation, and increased apoptosis [38]. A report revealed that murine peritoneal macrophages incubated with urban PM could induce inflammatory cytokines release, resulting in phenotype changes (M1/M2 polarization), cell injury, and decreased engulfment function [39]. Other reports had shown that PM exposure induced lung macrophage differentiation into a more pro-inflammatory subtype (M1-phenotype) rather than an anti-inflammatory subpopulation (M2-phenotype) [40,41], which attenuates the phagocytic activity against bacterial invasion, and probably led in more susceptible to pulmonary infection [39,40,41,42,43].

PM could also induce cAMP secretion and activate further downstream pathways, resulting in the enhancement of PM-mediated IL-6 release in murine alveolar macrophages (MH-S) and human alveolar macrophages [44]. In addition, administration of albuterol, a β_2_AR agonist, enhanced PM-induced IL-6 release in human alveolar macrophages and MH-S cell lines, while the alveolar macrophages from Adrb2^−/−^ mice incubated with albuterol showed a decreased PM-mediated IL-6 response [44]. These findings suggest that PM-mediated IL-6 release was dependent on the activation of β_2_AR, encoded by the Adrb2 gene. Moreover, PM was also shown to activate the microparticles and cause intracellular calcium release, and to enhance tissue factor function in HUVECs and peripheral blood mononuclear cells (PBMCs) [45]. The activation of tissue factors would further trigger blood coagulation via the TF-FVII complex, leading to thrombus formation.

In in vitro studies of venous blood from rodent models, incubation with DEP rapidly induced platelet activation and platelet aggregation in a dose-dependent manner [46,47,48,49]. This effect was emphasized in diabetic mice, which were more vulnerable to thrombotic complications [49]. In addition, incubation of HUVECs with DEP revealed decreasing tPA and PAI-1 activity [50]. These findings suggested that both DEP and PM induced thrombosis by activating the tissue factor pathway and enhancing platelet aggregation, as well as inhibiting the fibrinolytic process, thus promoting blood clot formation and thrombosis.

The different types of particles also had differing effects on outcomes. Positively-charged amine-particles could enhance platelet function, as indicated by the shortening of PFA100 closure time in both 60-nm UFP and 400-nm amine-polystyrene particles. In contrast, negatively-charged carboxylated UFP and unmodified UFP did not affect platelet function [51]. This means that the character of each particle also plays a role in PM-induced platelet aggregation. A summary of these in vitro reports on the effects of PM on inflammation, oxidative stress, adhesion molecules, coagulation, and cell proliferation is shown in Table 1.

## 4. The Effects of Particulate Matter on Inflammation, Oxidative Stress, and the Coagulation System: Reports from In Vivo Studies

Consistent with in vitro reports, in vivo studies showed that the β_2_-adrenergic receptor (β_2_AR) in the macrophages encoded for by the Adrb2 gene was the key receptor that modulates PM-induced inflammation and thrombosis [44]. Adrb2-knockout mice had increased the PM-mediated IL-6 release, whereas this effect was blunted in specific Lyms-Cre Adrb2^flox/flox^ mice together with a decrease in thrombus formation and tissue factors [44]. In addition, Lyms-Cre Adrb2^flox/flox^ mice pretreated with a β_2_AR agonist, Formoterol, showed that these mice which lack the Adrb2 gene in the macrophages had blunted IL-6 and TF response, in comparison to Adrb2^flox/flox^ mice after PM exposure [44]. Furthermore, PM exposure in mice with the depleted alveolar macrophages resulted in a decrease in both IL-6 release and thrombus formation [52]. These findings supported the results from in vitro studies which investigated PM induced inflammation through activation of the sympathetic nervous system via β_2_AR signaling in the macrophages. This further activated the release of IL-6, TNF-α, and TF leading to the thrombus formation [44,52,53,54,55,56,57].

PM may also induce oxidative stress, resulting in DNA damage [55,57]. Sirtuin 1 (Sirt1), the NF-ĸB regulatory gene, plays a key role in controlling the effects of PM-mediated inflammation [53]. Sirt1-knockout mice showed a higher level of inflammatory cytokines such as IL-6 and TNF-α after being exposed to PM_2.5_ [53], whereas a blunted inflammatory response was observed in the IL-6 knockout mice [52,56]. These findings emphasized the mechanistic links between the inhalation of PM and the stimulation of the sympathetic nervous system via β_2_AR, resulting in IL-6 release, systemic inflammation, and thrombosis, these processes being regulated by Sirt1. In addition, PM also induced oxidative stress in the rodent models. An increase in nitric oxide synthase (NOS) and heme oxygenase-1 (HO-1), and a decrease in catalase function in response to PM exposure were observed, together with an increase in the antioxidative response indicated by increase in glutathione (GSH) and a decrease in ascorbate level [55,57]. This increased anti-oxidative effect could be due to the compensation of the PM-mediated oxidative effects in those models.

PM_2.5_ may induce alveolar wall thickening and also enhance adhesion molecule and TF function [54]. PM_2.5_ also damaged vascular endothelial cells, resulting in TF release, which further activated the coagulation cascade and enhanced thrombus formation [53,54,55,56]. Intratracheal instillation of road tunnel dust in C57BL/6 mice could trigger TF release more extensively than in the mice exposed to urban dust, which was more pronounced at 48 h than at four hours after exposure [58]. These findings indicated that PM induced vascular injury and activated the TF pathway, with this process dependent on the composition of the PM and occurring in a time-dependent manner.

A study in hamsters revealed that PM_2.5_ exposure showed lower vWF levels, despite higher markers of vascular injury and vascular adhesion molecules [54]. This condition is characteristic of disseminated intravascular coagulation (DIC) which demonstrated obvious effects of PM_2.5_ on the prothrombotic state including evidence of extensive microvascular thrombi, decreased vWF, decreased coagulation factor levels, and prolonged clotting time, which resulted from a combination of the action of many clotting factors and vWF [54]. However, there are inconsistent reports. In a study in mice exposed to PM_2.5_ and PM_10_, no significant changes in vWF secretion and white blood cell (WBC) influx in the lungs and plasma were observed [59]. Although these two studies performed similar repetitive PM exposure for the same duration, these inconsistent findings could result from the differences in dose of PM exposure and in the species used. In the case of PM-mediated thrombosis, it has been shown that PM exposure could induce platelet activation, platelet function [59,60], and coagulation factors such as TF, and Factors II, VIII, and X [52,54]. Activation of platelets, TF, and coagulation cascades would further accelerate thrombin generation and induce a prothrombotic state [44,51,52,53,54,56,58,60]. One report revealed more thrombus formation at 48 h than at four hours after exposure, indicating that PM-accelerated thrombosis could be time-dependent [58].

The effect of PM on the fibrinolytic system remains controversial. Most studies reported that PM exposure alleviated fibrinolysis as a result of increased PAI-1 and decreased tPA mRNA expression, which caused the suppression of fibrinolytic activity and ultimately promoted a prothrombotic state [53,55,56]. A study which reported outcomes contradictory to these found that repetitive PM exposure increased tPA, reflecting the enhancement of fibrinolytic function [54]. It is possible that prolonged or chronic repetitive exposure to PM might induce extensive blood clots, leading to increased fibrinolytic activity as a compensatory response. However, further studies focusing on the fibrinolytic pathway are needed to enable us to understand the balance of each fibrinolytic factor and the ultimate effects of PM on fibrinolysis.

The effect of PM exposure on the blood cell count was inconclusive due to the conflicting data. Although PM was shown to increase the number of red blood cells (RBC) and hemoglobin (Hb) levels in mice [59], another study showed no significant impact [55]. The discordant results could be due to the difference in the duration of PM exposure, or the repetitive or prolonged duration of exposure, which might have more impact on PM-mediated changes than a single exposure or for a shorter duration. No WBC count changes were associated with PM exposure [55,59]. The effects of PM on platelet count were also uncertain since there were conflicting data among reports [52,54,55,59]. The possible explanation is that there were differences in particle type, exposure dose and/or duration, and in the severity of systemic activation of the coagulation pathway, especially the induction of DIC in those studies. Nevertheless, these in vivo studies emphasized that PM could induce lung injury via the stimulation of inflammation and oxidative stress, which further activates platelets and the coagulation system, leading to a hypercoagulable state. A summary of in vivo reports on the effects of PM on inflammation, oxidative stress, and the coagulation system is shown in Table 2.

## 5. The effects of Diesel Exhaust Particles (DEP) on Inflammation, Oxidative Stress, and Coagulation Systems: Reports from In Vivo Studies

Previous studies into acute and prolonged DEP exposure showed adverse cardiopulmonary impact [19,61,62,63,64,65]. Pulmonary exposure to DEP stimulated local and systemic inflammatory responses, including the release of IL-6 [48,50], TNF-α [47,66], IL-1β [47], and CRP [66]. DEP also triggered mast cells, resulting in histamine release [46,60,67,68]. Most studies consistently found that intratracheal instillation of DEP induced an influx of inflammatory cells such as WBC, neutrophils, and macrophages into the pulmonary system [46,48,50,66,67,68]. Although intravenous injection (IV) of DEP did not initiate an inflammatory response in bronchoalveolar lavage fluid (BALF), administration of DEP intravenously may activate systemic platelet-monocyte aggregation and intravascular thrombus formation [50]. These findings implied that DEP exposure could also activate the systemic coagulation pathway, which is independent of pulmonary inflammation. Moreover, DEP exposure could enhance the production of reactive oxygen species and decrease the antioxidant effect in Tuck-Ordinary (TO) mice [47,48]. Diabetic mice were also shown to be more susceptible to DEP and had higher concentrations of reactive oxygen species than non-DM mice [49].

As regards the coagulation and fibrinolytic systems, DEP exposure did not affect vWF in normal hamsters and mice [49,66,68]. However, elevated vWF levels were found in DM mice exposed to DEP [49]. These findings suggested that DEP exposure could promote a prothrombotic state via an increase in vWF level in DM mice, and imply that DM mice may be more susceptible to DEP than non-DM mice. Furthermore, in rodent models DEP exposure promoted thrombus formation [46,67,68], shortened PFA100 closure time which reflected an increase in platelet function [46,67], shortened time to thrombosis [47,48,49,50,66,67] and inhibited fibrinolysis [49,50,66]. Formation of a thrombus without the counteracting balance from the fibrinolytic system would lead to significant thrombosis.

DEP exposure had some impact on blood cell count, including RBC, Hb, hematocrit (Hct), WBC, and platelets. Some studies reported an elevated number of red cells, Hb, Hct, and WBC [47,48]. Following a lower dose of DEP, significant elevation of WBC was found only in DM-mice, but not in non-DM [49], probably because DM mice were more susceptible to DEP than non-DM mice. Currently, the information pertinent to the impact of DEP on platelet counts remained controversial. In TO mice, decreased platelet counts at 4 and 18 h after exposure to DEP were reported [48,66]. In contrast, another report showed an elevation in platelet numbers at 24 h after DEP exposure [47]. However, several studies reported no significant alteration in the platelet number in association with DEP exposure in the hamsters [46,67]. These inconsistent results suggested that platelet response might exhibit some dynamic changes over time after DEP exposure, and different species could have different responses. The changes and fluctuations of blood cell parameters in association with DEP exposure need to be investigated further. A summary of in vivo reports on the effects of DEP on inflammation, oxidative stress, and coagulation system is shown in Table 3.

## 6. The Effects of Particulate Matter on Inflammation and the Coagulation System: Reports from Clinical Studies

The cumulative evidence from clinical studies demonstrates that inhalation of ambient PM could activate systemic inflammation [69,70,71,72]. A summary of clinical studies on the effect of PM on inflammation, adhesion molecules, and thrombosis is shown in Table 4 [69,70,71,72,73,74,75,76,77,78]. Levels of inflammatory biomarkers such as MIP-1α, β, MCP-1, and sRAGE have been shown to be elevated after acute exposure to PM_2.5_ [69,70]. In contrast, other studies showed no significant association between either DEP or PM_2.5_ and other inflammatory cytokines including IL-6, IL-10, and IL-1β [69,71]. These inconsistent findings could result from the differences in production mechanism of each cytokine to PM exposure. However, to gain more insight into the PM-mediated inflammatory response, further clinical studies are needed.

Currently, knowledge around the effect of PM on CRP remains inconclusive. Studies in short-term exposure to ambient PM in young and elderly patients with coronary arterial disease (CAD) showed a significant association between CRP and elevated PM [72]. However, short-term and intermediate-term exposure to PM did not show a correlation with CRP in healthy elderly people, or in elderly patients at risk of cardiovascular disease (CVD) [70,73]. It has been reported that younger people have lower CRP levels than elderly individuals [74]. This probably explains why the PM-induced elevation of CRP in younger adults is more obvious than in elderly people. Although high-sensitivity CRP (hsCRP) is more sensitive when it comes to the detection of systemic inflammation, PM-mediated change in hsCRP in the elderly was not found after short-term exposure to urban PM [75].

Fine PM may affect endothelial cells, resulting in vascular endothelial injury and release of adhesion molecules. A study in healthy elderly individuals exposed to ambient PM_2.5_ in Boston showed that elevated ICAM-1 and VCAM-1 were significantly directly associated with the level of PM_2.5_ at many time courses from the four hour to 28 days moving average [73]. However, another experimental study in healthy young adults and metabolic syndrome patients did not show any effects of PM-mediated adhesion molecule release after inhalation exposure to DEP for 2 h [71]. The possible explanations of these discrepancies could be due to differences in the composition of the pollution, dosage, and duration of PM exposure.

PM exposure could induce many aspects of the prothrombotic state. Several studies reported that P-selectin and platelet factor 4 (PF4), reflecting platelet activation, had a positive association with PM exposure in healthy young adults and patients with coronary arterial disease [69,72,76]. Evidence from in vitro studies revealed that DEP could enhance platelet aggregation in a dose-dependent manner [47,48,49], especially in venous blood from DM mice [49]. Nevertheless, a clinical study in the healthy individuals exposed to DEP or in diabetic patients exposed to ambient PM in New York showed no significant change in the PM-mediated platelet counts and function from the platelet aggregation test [77,78]. These conflicting results could be due to the level of the ambient PM in this clinical report being inadequate to cause significant changes in the clinical outcomes.

**Table 4:** The effects of particulate matter on inflammation, oxidative stress, adhesion molecules, and thrombosis: Evidence from clinical studies.

Models	Exposure/Method	Results			Interpretation	References
		Inflammation	Coagulation & Adhesion Molecules	Blood Parameters		
Healthy young adults (N = 16) Mean age 25 y	Inhalation of # Petrodiesel exhaust (PDE) # Mixture of biodiesel 30% and 70% of petrodiesel from rapeseed methyl ester (RME30) # Biodiesel 100% from rapeseed methyl ester (RME100) For 1 h Evaluation at 2, 4, 8, 24 h after exposure		↔ tPA ↔ thrombus formation	↔ Hb, WBC, platelet	Inhalation exposure of biodiesel formulation (RME30, RME100) did not alter coagulation and blood cell parameters in comparison to PDE.	[77]
# Healthy adults (N = 15) Mean age: 28 y # Metabolic syndrome patients (N = 17) mean age 40 y	Inhalation of # DEP # Filtered fresh air (control) for 2 h Evaluate at pre-exposure, 7 and 22 h after exposure	↔ MMP-9 ↔ IL-1β ↔ IL-6 ↔ IL-10	↔ E-selectin ↔ ICAM-1, VCAM-1	# Both healthy and metabolic syndrome subjects: ↑ Hct ↔ Hb, RBC ↑ platelet ↔ WBC	Short-term DEP exposure resulted in hemoconcentration & thrombocytosis but did not affect inflammatory response and endothelial cell activation in both healthy and metabolic syndrome subjects.	[71]
Healthy adults (N = 73) Mean age 23.3 y	Ambient air pollution in an urban area, Beijing, China Evaluate the PM level at 1, 2, 3, 5, and 7 d MA	# PM_2.5_: ↑ sRAGE ↑ MIP-1α, β ↔ IL-1β, CRP # BC: ↑ sRAGE ↑ MIP-1α, β ↔ IL-1β, CRP	# PM_2.5_: ↑ P-selectin ↑ sCD40L ↔ PT ↑ FDP # BC: ↑ P-selectin ↔ sCD40L ↓ PT ↑ FDP		Exposure to higher ambient air pollution was associated with increased inflammatory biomarkers and heightened thrombogenicity.	[69]
Healthy young adults (N = 125) Mean age 24.2 y	Ambient air pollution in Beijing, China # Pre-Olympic period # During Olympics (Strict pollution control) # Post-Olympics period Evaluate the PM level at 1, 12, 24, 48, 96 h		# PM_2_.5, EC: ↑ P-selectin, sCD40L ↑ fibrinogen, VWF # OC: ↑ P-selectin, ↔ sCD40L ↑ fibrinogen, VWF # Pre- vs. During Olympic period: ↓ P-selectin, sCD40L ↔ fibrinogen ↓ VWF # During vs. post-Olympic period: ↑ P-selectin, ↔ sCD40L ↔ fibrinogen ↔ VWF	# PM, OC, EC: ↔ WBC # Pre- vs. During Olympic period: ↔ WBC # During vs. post-Olympic period: ↔ WBC	The restricted air pollution control markedly reduced PM, which was associated with decreased platelet activation and prothrombotic state. The alteration of PM level did not affect WBC count.	[76]
Elderly individuals with either CVD, or COPD and healthy individuals (N = 47) Mean age 78 y	Ambient air pollution fine PM outside of each individuals’ homes Seattle, WA, USA Evaluation of the PM level at the zero d and 1 d MA	↔ CRP ↑ MCP-1	↔ fibrinogen ↔ D-dimer		The effects of low ambient levels of PM on inflammation or thrombosis were not significant in elderly individuals.	[70]
Healthy elderly (N = 704) Mean age 73.2 y	Ambient Air Pollution in Boston, USA Evaluate the PM level at 4, 24 h, 3, 7, 14, 21, and 28 d MA	# PM_2.5_: ↔ CRP # BC: ↔ CRP	# PM_2.5_: ↑ ICAM-1, VCAM-1 ↔ fibrinogen # BC: ↑ ICAM-1, VCAM-1 ↑ fibrinogen		Short-term (1–3 d MA), and intermediate-term (7–28 d MA) exposure to traffic-related air pollution were associated with alteration of adhesion molecules, reflecting acute inflammatory and endothelial responses.	[73]
Adult patients undergoing cardiac catheterization due to stable IHD or ACS (N = 135) Mean age 61.4 y	Ambient air pollution Rochester, NY, USA Evaluate the PM level at 1, 12, 24, 48, 72, and 96 h MA	# PM_2.5_: ↑ CRP # Delta-C, AMP: ↔ CRP # BC: ↑ CRP # UFP: ↔ CRP	# PM_2.5_, Delta-C, BC: ↑ PF4 ↑ fibrinogen ↔ VWF, D-dimer # AMP ↑ PF4 ↑ fibrinogen ↔ P-selectin ↔ VWF, D-dimer # UFP: ↓ PF4 ↑ fibrinogen ↔ P-selectin ↔ VWF, D-dimer		The high PM was generally associated with an increase in biomarkers of systemic inflammation and coagulation.	[72]
Patients with CAD or at least two CVD comorbid diseases (HT, DM, hyperlipidemia) (N = 61) Mean age 62.3 y	Ambient air pollution In an urban area, Taipei City, Taiwan Evaluate the PM level at 1 to 3 d MA	# PM_2.5_, OC, EC: ↔ hsCRP	# PM_2.5_: ↔ fibrinogen ↔ D-dimer # OC, EC: ↔ fibrinogen ↑ D-dimer		Short-term exposure (1–3 d) to urban pollution triggered systemic inflammatory and thrombotic response in high-risk CVD patients.	[75]
DM type II (N = 30) Mean age 56.5 y	Acute exposure to ambient PM in Rochester, NY, USA Evaluate the PM level at 1, 12, 24, 48, 96 h		# PM_2.5_, AMP: ↔ TBXB2 ↔ ADP-, and collagen-induced platelet aggregation # UFP: ↔ TBXB2 ↔ ADP-induced platelet aggregation ↓ collagen-induced platelet aggregation at 48–96 h MA # BC: ↓ TBXB2 at 48–96 h MA ↔ ADP-, and collagen-induced platelet aggregation		High UFP levels were associated with reduced platelet response, whereas PM_2.5_, AMP, and BC resulted in a trend of increased platelet aggregation.	[78]

ACS: acute coronary syndrome, ADP: adenosine diphosphate, AMP: accumulation mode particles, BC: black carbon, CAD: coronary artery disease, COPD: chronic obstructive pulmonary disease, CRP: C-reactive protein, CVD: cardiovascular disease, d: days, DEP: diesel exhaust particles, DM: diabetes mellitus, E-selectin: endothelial cell adhesion molecule, EC: elemental carbon, FDP: fibrin degradation product, h: hours, Hb: hemoglobin, Hct: hematocrit, hsCRP: high sensitivity C-reactive protein, HT: hypertension, ICAM-1: intercellular adhesion molecule, IHD: ischemic heart disease, IL-1β: interleukin-1β, IL-6: interleukin-6, IL-10: interleukin-10, MA: moving average, MCP-1: monocyte chemoattractant protein-1, MIP-1: macrophage inflammatory protein-1, MMP-9: matrix metallopeptidase 9, OC: organic carbon, PF4: platelet factor 4, PM: particulate matter, PM_2.5_: particulate matter in diameter <2.5 µm, PMN: polymorphonuclear cells, PT: prothrombin time, RBC: red blood cells, sCD40L: soluble CD40 ligand, sRAGE: soluble receptor for advanced glycation end products, TBXB2: thromboxane B2, tPA: tissue plasminogen activator, UFP: ultrafine particles, VCAM-1: vascular cell adhesion molecule, VWF: von Willebrand factor, WBC: white blood cells, y: years.

There is limited data regarding the effect of PM on platelet counts in clinical studies. One study reported increased platelet counts in healthy adults and metabolic syndrome patients after inhalation of DEP for two hours [71]. Another report in healthy individuals exposed to DEP for one hour showed no significant difference in the platelet counts between exposure to petrodiesel and biodiesel [77]. A study in young healthy subjects showed that ambient PM had a significant positive correlation with the vWF level [76]. Another paper from a study in elderly patients with CAD reported no significant change in vWF following the PM exposure [72]. It is possible that since elderly individuals usually have higher vWF at base line [79,80,81], the PM-mediated effect may not result in any significant increment.

In terms of the coagulation pathway, there are only a few clinical studies which have investigated the PM-affected coagulation test. One report in young healthy adults and another report in elderly patients with coronary arterial disease described elevated fibrinogen levels in association with high ambient PM in Beijing and New York, respectively [72,76], while other reports in healthy elderly and patients with cardiovascular disease in various centers including Boston, Seattle, and Taipei found no significant correlation [70,73,75]. One possible explanation is that fibrinogen is a positive acute phase reactant protein [82,83], and the degree of systemic inflammation resulting from PM exposure may increase the fibrinogen level. Moreover, the ambient pollution in each area may contain different levels and types of toxic substances, and therefore the results may differ between research centers.

Another study revealed that PM was significantly associated with the increased fibrin degradation product (FDP), reflecting the occurrence of a blood clot and clot degeneration [69]. Conversely, other studies reported no changes in the alteration in D-dimer level, thrombus formation, or in coagulation time [69,70,72,75,77]. To date, no clinical data has been reported on the effect of PM on fibrinolysis. Further investigation is needed to explore this aspect of pollution.

There are only a few clinical studies which have focused on the impact of PM on blood cell parameters. Two reports revealed no significant changes in the RBC and Hb levels in association with exposure to PM [71,77], but one study reported elevated Hct levels, which is probably due to hemoconcentration [71]. No significant changes in the WBC count were demonstrated [71,76,77]. To clarify the significant impact of PM exposure on blood cell parameters as well as its association with clinical outcomes, future well-designed prospective studies are needed, with serial blood cell counts pre- and post-PM exposure, together with collection of data specific to various inflammatory biomarkers compared to the occurrence of clinical thrombosis or other clinical outcomes. The clinical studies regarding PM-mediated effects emphasized that PM could activate an inflammatory response, induce vascular endothelial injury, and activate the coagulation system, leading to a prothrombotic state, which is consistent with findings from the in vitro and in vivo studies.

## 7. In Vitro Interventional Reports on the Effects of Particulate Matter on Inflammation, Oxidative Stress, and Coagulation System

A previous in vitro and in vivo study demonstrated that β_2_AR encoded for by the Adrb2 gene plays a significant role in the PM-induced inflammation and thrombosis [44]. Consistent with this idea, the adding of propranolol, a βAR antagonist, was shown to alleviate the PM-mediated IL-6 effect in wild-type alveolar macrophage and MH-S cell lines [44]. These findings suggested that the blockade caused by the β_2_AR blocker had beneficial effects on reduction of the PM-induced adverse inflammatory response.

Previous reports also stated that PM exposure induced oxidative injury, which further activates the inflammatory response [84,85,86]. These findings suggest that anti-inflammatory and antioxidant agents may help prevent the adverse effects of PM on the prothrombotic state. It has been shown that the antioxidants Mito-Q or EUK-134 could block the release of PM-induced IL-6 and cAMP [44]. Administration of the phosphodiesterase inhibitor which blocked phospholipase C function was also shown to inhibit PM-induced microparticle release, suggesting that PM-mediated inflammation occurred through the activation of the phospholipase C, which further enhanced the prothrombotic state by the release of microparticles [45]. Emodin, which is a strong plant-based anti-inflammatory and antioxidative agent, could alleviate the thrombotic process by inhibiting platelet aggregation, and prolonging clotting time [47]. However, Thymoquinone, another plant-based agent with broad anti-inflammatory properties, failed to demonstrate any protective effects as regards platelet aggregation [48]. These discordant effects could possibly be due to the additional antioxidative effect of Emodin, which was not found in Thymoquinone. Nevertheless, most in vitro studies demonstrated that βAR antagonists, anti-inflammatory and antioxidant agents provided beneficial effects with regard to the attenuation of PM-mediated thrombosis. A summary of in vitro interventional reports on the effects of PM on inflammation, oxidative stress, and coagulation following exposure to PM is shown in Table 5.

## 8. In Vivo and Clinical Interventional Reports on the Effects of Particulate Matter on Inflammation, Oxidative Stress, and the Coagulation System

In mouse models, a study using either Reserpine, a chemical sympathectomy agent, or Propranolol, a βAR antagonist, demonstrated that blocking sympathetic effects could alleviate the PM-induced IL-6 response and reduce thrombus formation [44]. These findings suggested that βAR blocking agents could attenuate a PM-mediated thrombosis.

TNF-α was identified as being the key mediator that regulates PM-mediated fibrinolytic changes [56]. Administration of etanercept, a TNF-α inhibitor, before PM exposure in mice could prevent the expression of PM-induced PAI-1; however, there was no impact on coagulation tests and clotting formation [56]. This emphasized that PM inhibited fibrinolysis through TNF-α signaling. Antihistamines may suppress the effects of PM-mediated pulmonary inflammation, and procoagulation [46,67,87,88,89,90,91,92]. Administration of diphenhydramine before DEP exposure resulted in reduced inflammatory cell influx and less thrombus formation in hamsters [67]. Anti-inflammatory agents such as dexamethasone and sodium cromoglycate also reduced pulmonary inflammation and histamine release, as well as decreasing thrombin generation in the hamsters [68]. Curcumin, a potent anti-inflammatory agent, was shown to exert benefits in regard to the reduction of inflammatory biomarkers and the prothrombotic state in TO mice [66]. In addition, other anti-inflammatory agents such as Emodin and Thymoquinone also prevented pulmonary inflammation, and oxidative stress and reduced thrombosis in mice exposed to DEP [47,48]. A positive outcome was reported from one clinical study which demonstrated that aspirin and fish oil, both anti-inflammatory agents alleviated PM-mediated platelet aggregation [78].

Findings from both in vivo and clinical studies supported the findings from in vitro studies that PM-mediated inflammation, oxidative stress, and thrombosis could be enhanced by administration of a β_2_AR-agonist, while these effects could be lessened by adding either a β_2_AR antagonist, TNF-α blocker, an antihistamine, or an anti-inflammatory/antioxidative agent. The summary of intervention reports on the effect of PM on the inflammation, oxidative stress, and thrombosis following PM exposure in in vivo and clinical studies is shown in Table 6.

## 9. Limitation of the Current Studies and Direction for Future

Although many previous studies explained the pathological effects of PM on inflammatory and oxidative stress responses associated with thrombosis, there were several gaps of knowledge to be explored. In the past decades, some reports directly focused on the effects of certain toxic components. However, most of the studies mainly evaluated the composite outcomes of PM that had different compositions of various organic and inorganic compounds. The different substances and the characteristics such as size, positive- or negative-charge properties, dose, and time-dependent effects had shown different outcomes. Additional in vitro and in vivo research should be done to determine which specific toxic components have certain characteristics, especially the dose-effects (transient or cumulative effects). Moreover, both in vitro and in vivo studies have shown PM-induced platelet aggregation and thrombosis, but there have been only a few studies on the coagulation- and adhesion-associated molecules, necessitating further study to fully comprehend the pathological effects of PM on the coagulation system. With this knowledge, the pathology of PM-induced blood clotting may be comprehended, and it may be possible to reduce the negative effects of the harmful components and halt the production and use of these poisonous substances.

## 10. Conclusions

Inhalation of ambient air pollution consisting of various toxic substances may induce pulmonary inflammation, and an oxidative stress response and induce the prothrombotic state through β_2_AR, IL-6, and the TNF-α signaling pathway. Lung injury together with the release of IL-6 would further enhance TF, activate platelets, vWF, and coagulation factor function, resulting in the generation of thrombi. PM has also been shown to inhibit the fibrinolytic pathway via TNF-α signaling. These processes would make blood clots more robust, and they may therefore eventually occlude the luminal vessels. The effects of PM exposure on inflammation, oxidative stress, adhesion molecules, and thrombus formation are summarized in Figure 1. Several interventional studies using anti-inflammatory, antioxidative agents and βAR antagonists showed some protective effects on PM-mediated inflammation and thrombosis. Further clinical investigations should be carried out to explore the role of anti-inflammatory agents and antioxidants as well as β_2_AR antagonists in the prevention of PM-mediated adverse clinical outcomes. However, to eradicate or attenuate the air pollution problem seems to be currently more important and challenging. Global intervention and social movements may be required to reduce the air pollution, protect fresh air, and alleviate the impact of inhaling ambient air pollution on our health.

## Figures and Tables

**Table 1:** The effects of particulate matter on inflammation, oxidative stress, adhesion molecules, and hemostatic changes: Evidence from in vitro studies.

Models	Exposure/Method	Results			Interpretation	References
		Inflammation and Oxidative Stress	Coagulation and Adhesion Molecules	Morphology and Cell Proliferation		
HUVECs	Vanadium oxide (V_2_O_5_) 3.12, 6.25, 12.5, 25 µg/cm^2^ for 1, 2, 3, 24, 48, 72 h	↑ ROS at 25 µg/cm^2^ ↑ NO at 25 µg/cm^2^ (time-dependent)	↑ VCAM-1 ↑ ICAM-1 ↑↑ PECAM-1	Morphology changed to fibroblast-like cells ↓ cell proliferation at 25 µg/cm^2^ ↑ annexin V, PI	Exposure to V_2_O_5_ induced oxidative stress, enhanced the expression of adhesion molecules, and affected cell survival by diminishing cell proliferation, shape changes, and apoptosis.	[38]
MH-S, Human alveolar macrophages	PM (SRM 1649a) 10 µg/cm^2^ for 24 h	# MH-S: ↑ IL-6 ↑ cAMP # Human alveolar macrophage: ↑ IL-6			β_2_AR encoding from the Adrb2 gene had an important role in PM-induced IL-6 release and activation of β_2_AR enhanced inflammatory response in both cell lines.	[44]
	PM (SRM 1649a) 10 µg/cm^2^ for 24 h Pretreated with β_2_AR agonist; albuterol 10^−7^ M	↑↑ IL-6				
Alveolar macrophages from Adrb2^−/−^ mice	PM (SRM 1649a) 10 µg/cm^2^ for 24 h Pretreated with β_2_AR agonist; albuterol 10^−7^ M	↓ IL-6				
HUVECs, PBMC	PM (SRM 1648a) 62.5, 125, 250 and 500 µg/mL for 1, 4, 24, 48 h	↑ MP (dose-, and time-dependent) ↑ intracellular Ca concentration		↑ TF activity	PM-induced MP release, which, mediated by calcium mobilization, resulted in the prothrombotic state in both cell lines.	[45]
HUVECs	DEP 10–150 µg/mL for 16 h ±thrombin stimulation 1 U/mL		# Without thrombin: ↓ tPA ↓ PAI-1 # With thrombin stimulation: ↔ tPA ↑ PAI-1		DEP enhanced arterial thrombus formation through decreased fibrinolytic function but did not affect cell survival.	[50]
Venous blood of hamsters	DEP (SRM 1650) 0.1, 0.5, 1, 5 µg/mL for 5 min		↓ PFA100 closure time, dose-dependent		DEP promoted thrombosis via platelet activation in a dose-dependent manner.	[46]
Venous blood of TO mice	DEP 1 µg/mL for 3 min		↑ platelet aggregation ↓ PT ↓ PTT		DEP promoted thrombosis by enhancing platelet aggregation and coagulation.	[47]
Venous blood of TO mice	DEP (SRM 2975) 0.1, 0.25, 0.5, 1 µg/mL for 3 min		↑ platelet aggregation at 0.5 and 1 µg/mL, dose-dependent		DEP promoted thrombosis by enhancing platelet aggregation.	[48]
Venous blood of TO mice Non-DM and DM mice	DEP (SRM 2975) 0.25, 0.5, 1 µg/mL for 3 min		# Non-DM mice: ↑ platelet aggregation at 1 µg/mL # DM mice: ↑↑ platelet aggregation, dose-dependent		DEP promoted thrombosis by enhancing platelet aggregation, which was more obvious in DM mice.	[49]
Venous blood of hamsters (Pfd Gold)	Polystyrene particles: # 60 nm UFP - unmodified - carboxylated - amined at 1 or 3 µg/mL # 400 nm: Amine-polystyrene particles at 3 or 9 µg/mL for 5 min		# Unmodified and carboxylated UFP: ↔ PFA100 closure time # Amine-UFP (60 nm): ↓ PFA100 closure time (3 µg/mL) # Amine-particles (400 nm): ↓ PFA100 closure time (9 µg/mL)		Exposure to positively charged UFP (60 & 400 nm) augmented platelet function, leading to thrombosis.	[51]

Ca: calcium, cAMP: cyclic adenosine monophosphate, DEP: diesel exhaust particles, DM: diabetes mellitus, HUVECs: human umbilical vein endothelial cells, h: hours, ICAM-1: intercellular adhesion molecule-1, IL-6: interleukin-6, MH-S: murine alveolar macrophage cell line, min: minutes, MP: microparticles, NO: nitric oxide, PAI-1: plasminogen activator inhibitor-1, PBMC: peripheral blood mononuclear cells, PECAM-1: platelet endothelial cell adhesion molecule-1, PFA100: platelet function analyzer-100, PI: propidium iodide, PM: particulate matter, PT: prothrombin time, PTT: partial thromboplastin time, ROS: reactive oxygen species, SRM: standard reference material, TF: tissue factor, TO mice: Tuck-Ordinary mice, tPA: tissue plasminogen activator, UFP: ultrafine particles, VCAM-1: vascular cell adhesion molecule-1, V_2_O_5_: Vanadium oxide.

**Table 2:** The effects of particulate matter on inflammation, oxidative stress, blood parameters, and hemostatic changes: Evidence from in vivo studies.

Models	Exposure/Method	Results			Interpretation	References
		Inflammation and Oxidative Stress	Coagulation and Adhesion Molecules	Blood Parameters		
Male Wistar rats 10–12 wk-old Cisplatin-induced AKI rats	Intratracheal instillation of Cerium oxide nanoparticles (CeO_2_ NPs) 1 mg/kg	Normal rats: # Kidney ↑ TNF-α, IL-6, GSH ↑ DNA damage # Lung tissue ↔ TNF-α, IL-6 ↓ catalase activity AKI rats: # Kidney ↑ TNF-α, IL-6, GSH ↑ DNA damage # Lung tissue ↑ TNF-α, IL-6 ↓ catalase activity			Pulmonary exposure to CeO_2_ NPs induced inflammation and oxidative stress, and damaged DNA in the kidney. These effects were enhanced in kidney injury models.	[57]
Male mice C57Bl6/j 8–12 wk-old IL-6^+/+^ IL-6^−/−^	Inhalation exposure to concentrated ambient particles (CAPs) from downtown Chicago for 8 h/d for 3 d Evaluate at 24 h after exposure	# IL-6^+/+^ vs. non-PM): Lung tissue ↑ IL-6/18s mRNA ↑ SP-B/18s mRNA BALF ↑ IL-6 ↑ TNF-α ↑ MCP-1 # IL-6^−/−^: Lung tissue ↓ IL-6/18s mRNA ↓ SP-B/18s mRNA BALF ↓ IL-6 ↔ TNF-α ↔ MCP-1	# IL-6^+/+^ vs. non- PM): Lung tissue ↑ TF/18s mRNA Plasma ↑ TAT complexes White adipose tissue ↑ PAI-1/18s mRNA # IL-6^−/−^: Lung tissue ↓ TF/18s mRNA Plasma ↓ TAT complexes White adipose tissue ↔ PAI-1/18s mRNA		Exposure to all types of PM could activate inflammatory response, coagulation system and inhibit fibrinolysis, resulting in a prothrombotic state. PM-induced coagulation through IL-6 production and blocking IL-6 signaling could alleviate the thrombotic process.	[56]
	Intratracheal instillation of urban PM (SRM1649a) 10, 100, 200 µg/animal Evaluate at 24 h after exposure	# IL-6^+/+^ vs. non- PM): BALF ↑ protein ↑ macrophage, PMN ↑ IL-6 (dose-dependent) ↑ TNF-α # IL-6^−/−^: BALF ↔ protein ↔ macrophage, PMN ↓ IL-6 ↔ TNF-α	# IL-6^+/+^ vs. non- PM): ↑ TF, ↑TF mRNA in lung tissue ↑ BALF D-dimer ↑ TAT complexes ↓ Bleeding time ↓ PT, ↓ PTT ↑ PAI-1/18s mRNA in the lung, adipose tissue ↑ PAI-1 in BALF # IL-6^−/−^: ↓ TF level, ↓TF mRNA in lung tissue ↓ BALF D-dimer ↓ TAT complexes ↔ PAI-1/18s mRNA in the lung, adipose tissue ↔ PAI-1 in BALF			
Male mice (C57BL/6) 8–12 wk-old	Inhalation exposure to concentrated ambient particles (CAPs) from downtown Chicago for 8 h/d for 3 d	↑ NE in the lung, BAT, adrenal gland ↑ IL-6 in BALF	↑ TAT complexes ↑ thrombus formation ↓ thrombotic occlusion time		Inhalation of PM caused catecholamine release and promoted IL-6-mediated thrombosis.	[44]
Adrb1^+/+^Adrb2^+/+^ Adrb1^−/−^Adrb2^+/+^ Adrb1^+/+^Adrb2^−/−^ Adrb1^−/−^Adrb2^−/−^	Intratracheal instillation of urban PM (SRM1649a) 200 µg/animal Evaluate at 24 h after exposure	BALF # Adrb1^+/+^Adrb2^+/+ (^vs. non-PM): ↑ IL-6 ↔ TNF-α, MCP-1 # Adrb1^−/−^ Adrb2^+/+^ (vs. non-PM): ↑ IL-6 ↔ TNF-α, MCP-1 # Adrb1^+/+^Adrb2^−/−^: ↓ IL-6 ↔ TNF-α, MCP-1 # Adrb1^−/−^Adrb2^−/−^: ↓ IL-6 ↔ TNF-α, MCP-1	Plasma # Adrb1^+/+^Adrb2^+/+ (^vs. non-PM): ↑ TAT complexes ↓ thrombotic occlusion time # Adrb1^−/−^ Adrb2^+/+^ (vs. non-PM): ↑ TAT complexes # Adrb1^+/+^Adrb2^−/−^: ↓ TAT complexes ↑ thrombotic occlusion time # Adrb1^−/−^Adrb2^−/−^: ↓ TAT complexes		β_2_AR encoded by the Adrb2 gene in alveolar macrophages was necessary for PM-induced upregulation of IL-6, and enhanced susceptibility to thrombotic events.	
Adrb1^+/+^Adrb2^+/+^ Adrb1^+/+^Adrb2^−/−^	Inhalation exposure to concentrated ambient particles (CAPs) from downtown Chicago for 8 h/d for 3 d	# Adrb1^+/+^Adrb2^−/−^: ↓ IL-6/18s mRNA	# Adrb1^+/+^Adrb2^−/−^: ↓ TAT complexes ↓ TF			
Lyms-Cre Adrb2^flox/flox^ mice (macrophage-specific deletion of β_2_AR) vs. Adrb2^flox/flox^	Inhalation exposure to concentrated ambient particles (CAPs) from downtown Chicago for 8 h/d for 3 d Pretreated with formoterol (long-acting β_2_AR agonist) 1 × 10^−5^ M via inhalation twice every 12 h	BALF # Adrb2^flox/flox^ without formoterol: ↑ IL-6 in BALF # Adrb2^flox/flox^ with formoterol: ↑↑ IL-6 # Lyms-Cre Adrb2^flox/flox^: ↓ IL-6 # Lyms-Cre Adrb2^flox/flox^ with formoterol: ↓ IL-6 (vs. Adrb2^flox/flox^) ↔ IL-6 (vs. without formoterol)	Plasma # Adrb2^flox/flox^ without formoterol: ↑ TAT complexes ↑ factor II, TF mRNA ↓ thrombotic occlusion time # Adrb2^flox/flox^ with formoterol: ↑ factor II, TF mRNA ↓ thrombotic occlusion time # Lyms-Cre Adrb2^flox/flox^: ↓ factor II, TF mRNA ↓ TAT complexes ↑ thrombotic occlusion time # Lyms-Cre Adrb2^flox/flox^ with formoterol vs. Adrb2^flox/flox^: ↓ factor II, TF mRNA ↑ thrombotic occlusion time vs. without formoterol: ↔ factor II, TF mRNA ↔ thrombotic occlusion time			
Male mice C57Bl6/j Old mice (20 mo-old) vs. Young mice (10 wk-old)	Inhalation of ambient PM_2.5_ and PM_10_ at the roadside tunnel for 25–26 d (A) tunnel-filtered (B) tunnel-exposed in urban roadside tunnel (C) control in clean facility	# Young mice (vs. non-PM): ↔ WBC in BALF # Old mice (vs. young mice) in clean air: ↑ WBC in BALF # Old mice (vs. young mice) with PM: ↔ WBC in BALF	# Young mice (vs. non-PM): ↔ lung vWF ↔ plasma vWF ↓ lung TM ↑ P-selectin ↔ PF4 # Old mice (vs. young mice) in clean air: ↑ lung VWF ↑ plasma VWF ↔ lung TM ↑ P-selectin ↔ PF4 # Old mice (vs. young mice) with PM: ↑ lung vWF ↔ plasma VWF ↔ lung TM ↔ P-selectin ↔ PF4	# Young mice (vs. non-PM): ↑ RBC, Hb ↑ platelets ↔ WBC # Old mice (vs. young mice) in clean air: ↑ RBC, Hb ↑ platelets ↑ WBC # Old mice (vs. young mice) with PM: ↔ RBC, Hb ↔ platelets ↔ WBC	Continuous inhalation of particulate matter air pollution triggered inflammatory response, and activated platelets, and endothelial cells. The older mice had higher inflammatory biomarkers at baseline, therefore the PM-mediated effects were not demonstrated in the old mice.	[59]
Male mice C57Bl6/j with spontaneous hypertension 11–12 wk-old	Intratracheal instillation particulate matter # Road tunnel dust (RTD): 0.3, 1, 3, and 10 mg/kg # Urban dust (EHC-93) from Environmental Health Center in Ottawa, Canada 10 mg/kg Evaluation of lung tissue at 4, and 48 h after PM exposure		# RTD (at 10 mg/kg): - at 4 h: ↑ TF ↑ thrombus formation - at 48 h: ↑ TF ↑↑ thrombus formation # EHC-93: - at 4 h: ↔ TF ↑ thrombus formation - at 48 h: ↑↑ TF ↑↑ thrombus formation		PM induced procoagulant activity in the lungs, via increased TF expression and aggravated thrombus formation.	[58]
Hamsters (Pfd Gold) 100–110 g	Intratracheal instillation of polystyrene particles: # 60 nm UFP -unmodified 500 μg/animal -carboxylated 500 μg/animal -amined 5, 50, 500 μg/animal # 400 nm: Amine-modified polystyrene particles 500 μg/animal Evaluation of BALF at 1 h after UFP exposure	# Unmodified and carboxylated UFP: ↔ PMN influx # Amine-UFP (60 nm): ↑ PMN influx (50 and 500 µg/animal) ↑ protein, histamine (500 μg/animal) # Amine-particles (400 nm): ↑ PMN influx ↑ BALF protein ↔ BALF histamine	# Unmodified and carboxylated UFP: ↔ thrombus formation # Amine-UFP (60 nm): ↑ thrombus formation (at 50 and 500 µg/animal) # Amine-particles (400 nm): ↔ thrombus formation		Exposure to both positively charged UFP (60 & 400 nm) resulted in inflammation in the respiratory tract, but only the UFP (60 nm) rapidly activated the clotting system within an hour, leading to thrombosis.	[51]
Hamster 100–110 g	Intratracheal instillation of polystyrene particles: # 60 nm UFP - unmodified 500 μg/animal - carboxylated 500 μg/animal - amined 5, 50, 500 μg/animal # 400 nm amined- polystyrene particles 500 μg/animal Evaluation of BALF at 1 h after UFP exposure	# Unmodified and carboxylated UFP: ↔ PMN influx # Amine-particles (60 nm and 400 nm): ↑ PMN influx (50 μg) ↑↑ PMN influx (500 μg)	# Unmodified and carboxylated UFP: ↔ thrombus formation # Amine-particles (60 nm): ↑↑ thrombus formation (50 μg) ↑ thrombus formation (500 μg) # Amine-particles (400 nm): ↔ thrombus formation		UFP induced pulmonary inflammation and promoted thrombosis, but the degree of lung inflammation did not show a correlation with the extent of thrombosis.	[60]
	Intratracheal instillation of DEP (SRM 1650) 5, 50, 500 μg/animal Evaluate at 1 h after UFP exposure	BALF ↑ PMN influx ↑ protein ↑ histamine (at 50 and 500 μg/animal)	↑ thrombus formation (50 μg) ↑↑ thrombus formation (500 μg) ↓ PFA100 closure time		DEP exposure activated platelet and thrombin generation, leading to thrombosis.	
Female mice (C57BL/6) 8–10 wk-old sex-age-matched Sirt1 +/+ Sirt1 −/− Sirt1 overexpression in WT mice (vs. WT mice)	Intranasal instillation of PM_2.5_ (SRM 8785) 100 µg/animal for 24 h	# Sirt1 +/+: ↑ lung NF-ĸB ↑ BALF albumin, PMN ↑ BALF TNF-α & IL-6 # Sirt1 −/−: ↑↑ lung NF-κB ↑↑ BALF albumin, PMN ↑↑ BALF TNF-α & IL-6	# Sirt1 +/+: ↑ lung fibrin formation ↓ TFPI ↑ TF ↑ lung PAI-1 ↔ plasma PAI-1 ↓ lung TM # Sirt1 −/−: ↑ ↑ lung fibrin formation ↓ ↓ TFPI ↑ TF ↑ ↑ lung PAI-1 ↔ plasma PAI-1 ↓↓ lung TM # Sirt1 overexpression: ↓ lung fibrin formation ↑ lung TM		PM_2.5_ exposure promoted pulmonary vascular injury and enhanced inflammation, coagulation, and inhibited fibrinolysis, which was regulated by Sirt1 and NF-κB pathways.	[53]
Male SD rats 8–12 wk-old	Intratracheal instillation of PM_2.5_ once every 3 d for 30 d Doses: - Low dose: 1.8 mg/kg - Middle dose: 5.4 mg/kg - High dose: 16.2 mg/kg PM_2.5_ was collected from central Beijing, China	↑ Alveolar wall thickening ↑ IL-6, IL-1β, CRP ↔ MCP-1	↓ Aortic valve peak blood flow ↑ thrombus formation ↑ TF ↑ TAT complexes ↑ Factor Xa ↑↑ D-dimer ↓ TM ↔ TFPI ↑ tPA ↓ vWF ↑ PT, PTT, TT ↔ fibrinogen ↑↑ ICAM-1, VCAM-1	↓ platelets	PM_2.5_ induced vascular endothelial injury, systemic inflammatory response, altered coagulation factors, anticoagulant pathway, and fibrinolytic system, resulting in the prothrombotic state, and DIC.	[54]
Male Wistar Kyoto (WKY) rats 12–15 wk-old	Intratracheal instillation of PM_2.5_ and PM_10_ from The Northern and Southern Mexico - Total fraction - Insoluble fraction - Soluble fraction (control) of each PM_2.5_ and PM_10_ 3.3 mg/kg Evaluation at 24 or 72 h after PM exposure	# Total fraction and insoluble fraction of PM_2.5_ & PM_10_: ↑ BALF cell count ↓ alveolar macrophages Lung tissue ↑ total protein, ↑ albumin, ↓ ascorbic acid ↑ MIP-2, TNF-α mRNA ↑ BALF MIP-2, TNF-α ↑ HO-1 ↑ LOX-1R, ↑ NOS	# Total fraction and insoluble fraction of PM_2.5_ & PM_10_: ↑ lung TF mRNA ↓ tPA mRNA ↑ PAI-1 mRNA	# Total fraction and insoluble fraction of PM_2.5_ & PM_10_: ↔ RBC, Hb, Hct, platelet, and WBC	Exposure to PM aggravated pulmonary inflammation and oxidative stress, as well as disruption in the procoagulant and fibrinolytic pathways of the lung.	[55]
Male mice (C57BL/6) 8–12 wk-old IL-6^+/+^ IL-6^−/−^ IL-6^+/+^ depleted alveolar macrophages	Intratracheal instillation of PM_10_ from ambient air in Düsseldorf, Germany 10 μg/animal for 24 h # Pretreated with Intratracheally instillation of liposomal clodronate 120 mg/animal for 48 h before PM exposure (Setting of WT mice depleted of alveolar macrophages)	BALF # IL-6^+/+^ vs. non-PM_10_: ↑ macrophage, PMN ↑ IL-6, TNF-α, IFN-γ ↔ MCP-1, IL-10, IL-12 # IL-6^−/−^ vs. non-PM_10_: ↑ macrophage, PMN ↔ IL-6 ↑ TNF-α ↔ MCP-1, IL-10, IL-12, IFN-γ # IL-6^−/−^ vs. IL-6^+/+^: ↓ IL-6 ↔ TNF-α, MCP-1, IL-10, IL-12, IFN-γ # IL-6^+/+^ depleted alveolar macrophages: ↓ macrophage ↔ PMN ↓ IL-6 ↔ TNF-α, MCP-1, IL-10, IL-12, IFN-γ	Plasma # IL-6^+/+^ vs. non-PM_10_: ↑ Factor II, VIII, X ↑ Fibrinogen ↓ Bleeding time ↓ PT, ↓ PTT ↓ thrombotic occlusion time ↑ TAT complexes # IL-6^−/−^ vs. non-PM_10_: ↔ Factor VIII ↔ Bleeding time ↔ PT, ↔ PTT ↔ thrombotic occlusion time ↔TAT complexes # IL-6^+/+^ depleted alveolar macrophages: ↓ Factor VIII ↑ Bleeding time ↑ PT, ↑ PTT ↓ TAT complexes ↑ thrombotic occlusion time	# IL-6^+/+^ vs. non-PM_10_: ↑ Platelet # IL-6^−/−^ vs. non-PM_10_: ↔ Platelet # IL-6^+/+^ depleted alveolar macrophages: ↓ Platelet	PM_10_ exposure-induced pulmonary inflammation, and IL-6 release. IL-6 was the key mediator, which enhanced coagulation factor function, resulted in shortening of clotting time, and led to thrombosis. Blocking either the macrophage function or IL-6 signal could alleviate PM-induced prothrombotic state.	[52]

AKI: acute kidney injury, BALF: bronchoalveolar lavage fluid, BAT: brown adipose tissue, β_2_AR: adrenergic receptor beta-2, CAPs: concentrated ambient particles, CeO_2_ NPs: Cerium oxide nanoparticles, CRP: C-reactive protein, d: days, DEP: diesel exhaust particles, DIC: disseminated intravascular coagulopathy, DNA: deoxyribonucleic acid, EHC-93: Environmental health center-93, GSH: glutathione, h: hours, Hb: hemoglobin, Hct: hematocrit, HO-1: heme oxygenase-1, ICAM-1: intercellular adhesion molecule-1, IL-1β: interleukin-1beta, IL-6: interleukin-6, IL-10: interleukin-10, IL-12: interleukin-12, IFN-g: interferon-g, LOX-1R: lectin-like oxidized low-density lipoprotein receptor-1, MCP-1: monocyte chemoattractant protein-1, MIP-2: macrophage inflammatory protein-2, mo: months, mRNA: messenger ribonucleic acid, NE: norepinephrine, NF-κB: nuclear factor-κB, NOS: nitric oxide synthase, PAI-1: plasminogen activator inhibitor-1, PFA100: platelet function analyzer-100, PF4: platelet factor 4, PM: particulate matter, PM_2.5_: particulate matter in diameter <2.5 µm, PM_10_: particulate matter in diameter <10 µm, PMN: polymorphonuclear cells, PT: prothrombin time, PTT: activated partial thromboplastin time, RBC: red blood cells, RTD: road tunnel dust, SD rats: Sprague-Dawley rats, SP-B: surfactant protein B, SRM: standard reference material, TAT complexes: thrombin-antithrombin complexes, TF: tissue factor, TFPI: tissue factor pathway inhibitor, TM: thrombomodulin, TNF-α: tumor necrotic factor-α, tPA: tissue plasminogen activator, TT: thrombin time, UFP: ultrafine particle, VCAM-1: vascular cell adhesion molecule-1, VWF: von Willebrand factor, WBC: white blood cells, wk: week, WT mice: wild type mice.

**Table 3:** The effects of DEP on inflammation, oxidative stress, blood parameters, and hemostatic changes: Evidence from in vivo studies.

Models	Exposure/Method	Results			Interpretation	References
		Inflammation and Oxidative Stress	Coagulation and Adhesion Molecules	Blood Parameters		
Male Wistar rats 175–275 g	Intratracheal instillation of # DEP (SRM 2975) 0.5 mg/animal # Black carbon (BC) 0.5 mg/animal # DQ12 quartz microparticles 0.125 mg/animal Evaluation at 2, 6, and 24 h after exposure	BALF # DEP: ↑ PMN influx ↑ IL-6 ↔ TNF-α, CRP # BC: ↑↑ PMN influx ↑ IL-6 ↔ TNF-α ↑ CRP # DQ12 quarts: ↑↑ PMN influx ↔ IL-6 ↔ TNF-α ↑ CRP	Plasma # DEP ↓ thrombotic occlusion time ↑ PAI-1 ↓ tPA ↓ tPA:PAI-1 ratio ↑ platelet-monocyte aggregation # BC, DQ12 quarts: ↔ thrombotic occlusion time ↑ PAI-1 ↓ tPA ↓ tPA:PAI-1 ratio ↔ platelet-monocyte aggregation		Pulmonary exposure of DEP caused lung inflammation and accelerated arterial thrombus formation through increasing platelet activation, and impaired fibrinolytic function, while IV injection of DEP promoted thrombosis, without evidence of pulmonary inflammatory response.	[50]
	Intravenous injection (IV) of DEP or BC 0.5 mg/kg Evaluation at 2, 6, and 24 h after exposure	# DEP: ↔ inflammatory cells in BALF ↔ BALF TNF-α, CRP ↔ plasma TNF-α, CRP, IL-6 # BC: ↔ inflammatory cells in BALF ↔ BALF TNF-α, CRP ↔ plasma TNF-α, IL-6 ↑ plasma CRP	# DEP: ↓ thrombotic occlusion time ↑ PAI-1 ↓ tPA ↓ tPA:PAI-1 ratio ↑ platelet-monocyte aggregation # BC: ↓ thrombotic occlusion time ↑ PAI-1 ↓ tPA ↓ tPA:PAI-1 ratio ↔ platelet-monocyte aggregation			
Hamsters (Pfd Gold) 100–110 g	Intratracheal instillation of DEP (SRM 1650) 5, 50, 500 µg/animal Evaluate at 1 h after PM exposure	BALF ↑ PMN influx ↑ protein, histamine, in a dose-dependent manner ↔ LDH	↑ venous thrombus formation, dose-dependent manner ↑ arterial thrombus formation ↓ PFA100 closure time	↔ platelet	DEP enhanced lung inflammation, platelet activation, and peripheral vascular thrombosis.	[46]
Hamsters 100–110 g	Intratracheal instillation of DEP (SRM 1650) 50 µg/animal Evaluation at 1, 6, 24 h after exposure	↑ BALF PMN influx, time-dependent manner ↑ BALF histamine ↑ plasma histamine	↑ thrombus formation ↓ PFA100 closure time	↔ platelet	Histamine involved in the process of DEP-induced lung inflammation and platelet activation led to a prothrombotic state.	[67]
Hamsters (Pfd Gold) 100–110 g	Intratracheal instillation of # DEP (SRM 1650) 50 µg/animal # Polystyrene particles 400 nm 500 µg/animal Evaluation at 24 h after exposure	# DEP: ↑↑ PMN influx # Polystyrene particles: ↑ PMN influx # DEP and polystyrene particles: ↑ histamine in BALF and plasma	# DEP and polystyrene particles: ↑ thrombus formation ↔ VWF		DEP triggered mast cell degranulation by histamine release and enhanced thrombus formation.	[68]
Male TO mice (HsdOla: TO) 10–12 wk-old DM vs. non-DM mice (Intraperitoneal injection of streptozotocin 200 mg/kg to induced Type 1 DM)	Intratracheal instillation of DEP (SRM 2975) 0.4 mg/kg Evaluation of plasma at 24 h after exposure	# Non-DM mice: ↔ CRP ↔ 8-isoprostane # DM mice: ↑ CRP ↑ 8-isoprostane	# Non-DM mice: ↔ thrombotic occlusion time ↑ PAI-1 ↔ VWF # DM mice: ↓ thrombotic occlusion time ↑↑ PAI-1 ↑ VWF	# Non-DM mice: ↔ WBC ↔ platelet # DM mice: ↑ WBC ↓ platelet	Particulate air pollution activated systemic inflammation, oxidative stress, hypoxemia, hepatotoxicity, coagulation, and interfered with fibrinolytic function, resulting in a procoagulant state. These results were more enhanced in DM mice.	[49]
Male TO mice (HsdOla: TO) 30–35 g	Intratracheal instillation of DEP (SRM 2975) 15 µg/animal on day 0, 2, 4, 6 Evaluation at 48 h after the last exposure	BALF: ↑ PMN influx ↑ macrophages ↑ TNF-α ↔ IL-6 Plasma: ↑ CRP ↑ TNF-α ↔ IL-6	↓ thrombotic occlusion time ↑ D-dimer ↑ PAI-1 ↔ VWF	↓ platelet	Repeated DEP exposure activated systemic inflammation, thrombotic events, and platelet aggregation.	[66]
Male TO mice (HsdOla: TO) 30–35 g	Intratracheal instillation of DEP (SRM 2975) 30 µg/animal Evaluation at 4, and 18 h after exposure	BALF ↑↑ PMN, macrophages ↑↑ IL-6 ↑↑ total protein ↓ superoxide dismutase	↑ IL-6 ↓ thrombotic occlusion time	↑ WBC ↓ platelet	DEP exposure-induced pulmonary inflammation, and enhanced platelet aggregation and thrombosis.	[48]
Male TO mice	Intratracheal instillation of DEP 1 mg/kg Evaluation at 24 h after exposure	↑ TNF-α ↑ IL-1β ↓ superoxide dismutase ↑ glutathione reductase	↓ thrombotic occlusion time	↑ Hb, Hct, RBC, WBC, platelet	DEP exposure activated inflammation, oxidative stress, and promoted thrombosis.	[47]

BALF: bronchoalveolar lavage fluid, BC: black carbon, CRP: C-reactive protein, DEP: diesel exhaust particles, DM: diabetes mellitus, h: hours, Hb: hemoglobin, Hct: hematocrit, IL-1β: interleukin-1beta, IL-6: interleukin-6, IV: intravenous injection, PAI-1: plasminogen activator inhibitor-1, PFA100: platelet function analyzer-100, PM: particulate matter, PMN: polymorphonuclear cells, RBC: red blood cells, SRM: standard reference material, TNF-α: tumor necrotic factor-α, TO mice: Tuck-Ordinary mice, tPA: tissue plasminogen activator, VWF: von Willebrand factor, WBC: white blood cells, wk: week.

**Table 5:** The effects of pharmacological interventions on inflammation, oxidative stress, and hemostatic changes following exposure to particulate matter: Evidence from in vitro studies.

Models	Exposure	Intervention	Results		Interpretation	References
			Inflammation and Oxidative Stress	Coagulation and Adhesion Molecules		
Human alveolar macrophages, MH-S	PM (SRM 1649a) 10 µg/cm^2^ for 24 h	# β_2_AR agonists; albuterol 10^−7^ M # βAR antagonist; propranolol 10 µM # Albuterol + propranolol Added 1 h after PM	↑ IL-6 ↓ IL-6 ↓ IL-6		Activation of β_2_AR enhanced PM-mediated IL-6 release, while βAR blockade inhibited the release of IL-6 in response to PM.	[44]
MH-S	PM (SRM 1649a) 10 µg/cm^2^ for 1 h	# An adenylyl cyclase activator, Forskolin 50 µM # Forskolin 50 µM and adenylyl cyclase inhibitor (SQ2253) 300 µM # Forskolin + PDE inhibitors; IBMX 1 µM # Forskolin + Aminophylline 10 µM # Antioxidant; Mito-Q # Superoxide dismutase/catalase mimetic Eukarion 134 (EUK-134) # Forskolin + EUK-134	# Forskolin: ↑↑ IL-6 ↑ cAMP # Forskolin + SQ2253: ↑ IL-6 # Forskolin + IBMX: ↑ cAMP # Forskolin + Aminophylline: ↑ cAMP # Mito-Q: ↓ IL-6 # EUK-134: ↓ IL-6 ↔ cAMP # Forskolin + EUK-134: ↓cAMP		PM exposure enhanced IL-6 release and activated systemic inflammation via adenylyl cyclase and CREB functions.	
CREB shRNA-transfected MH-S cells p65-shRNA-transfected MH-S cells	PM (SRM 1649a) 10 µg/cm^2^ for 1 h	# Albuterol 10^−7^ M for 1 h	↓ IL-6			
HUVECs, PBMC	PM (SRM1648a) 500 µg/mL for 1 h	# Phospholipase C inhibitor (U73122) 1 µM for 30 min	↓ MP		PM-induced MP release was mediated through phospholipase C.	[45]
Venous blood of TO mice	DEP (SRM 2975) 1 µg/mL for 3 min	# Thymoquinone 0.1 mg/mL for 3 min		↔ platelet aggregation	Thymoquinone did not prevent DEP-induced platelet aggregation.	[48]
Venous blood of TO mice	DEP 1 µg/mL for 3 min	# Emodin 1 µg/mL for 3 min		↓ platelet aggregation ↑ PT ↑ PTT	Emodin prevented the effects of DEP-induced platelet aggregation and thrombosis.	[47]

β_2_AR: β_2-_adrenergic receptor, βAR: β-adrenergic receptor, cAMP: cyclic adenosine monophosphate, CREB: cAMP response element-binding protein, DEP: diesel exhaust particles, h: hours, HUVECs: human umbilical vein endothelial cells, IBMX: 3-isobutyl-1-methylxanthine, IL-6: interleukin-6, MH-S: murine alveolar macrophage cell line, min: minutes, MP: microparticles, PBMC: peripheral blood mononuclear cells, PDE inhibitor: phosphodiesterase inhibitor, PM: particulate matter, PT: prothrombin time, PTT: activated partial thromboplastin time, SRM: standard reference material, TO mice: Tuck-Ordinary mice.

**Table 6:** The effect of pharmacological interventions on inflammation, oxidative stress, blood parameters, and hemostatic changes under particulate matter condition: Evidence from in vivo and clinical studies.

Models	Exposure	Intervention	Results			Interpretation	References
			Inflammation and Oxidative Stress	Coagulation and Adhesion Molecules	Blood Parameters		
Male mice (C57BL/6) 8–12 wk-old	Intratracheal instillation of urban PM (SRM1649a) 200 µg/animal Evaluated at 24 h after exposure	Pretreated with # Vesicular monoamine transporter: Reserpine (chemical sympathectomy) # Propranolol 3 mg/kg IP q 8 h for 48 h	↓ NE in BALF, BAT, adrenal gland, lung ↓ IL-6 in BALF ↓ IL-6 in BALF	↓ plasma TAT complexes ↓ plasma TAT complexes ↑ thrombotic occlusion time		Blocking of the sympathetic nervous system and β_2_AR signaling alleviated IL-6 release, lung inflammation, and reduced thrombosis.	[44]
Male mice C57Bl6/j 8–12 wk-old	Inhalation exposure to concentrated ambient particles (CAPs) from downtown Chicago for 8 h/d for 3 d Evaluated at 24 h after exposure	Pretreated with TNF-α inhibitor, Etanercept 10 mg/kg IP. 3 days before, and on the first day of exposure to CAPs		↓ PAI-1/18s mRNA		Blocking TNF-α could promote normal fibrinolytic function, but not alter the PM-induced clotting formation.	[56]
	Intratracheal instillation of urban PM (SRM1649a) 200 µg/animal Evaluate at 24 h after exposure	Pretreated with TNF-α inhibitor, Etanercept 10 mg/kg IP. 3 days before, and on the first day of exposure to PM		↔ Bleeding time ↔ PT, ↔ PTT ↔ TAT complexes ↓ PAI-1/18s mRNA ↓ PAI-1 in BALF			
Hamsters 100–110 g	Intratracheal instillation of DEP (SRM 1650) 50 µg/animal For 1, 3, 6, 24 h after exposure	Pretreated with Antihistamine: Diphenhydramine IP 30 mg/kg for 1 h	BALF ↓ cell count, PMN influx ↓ histamine plasma ↓ histamine	↓ thrombus formation ↑ PFA100 closure time		Pretreatment with diphenhydramine reduced the effects of DEP-induced pulmonary inflammation and peripheral thrombosis.	[67]
Hamsters (Pfd Gold) 100–110 g	Intratracheal instillation of DEP (SRM 1650) 50 µg/animal Evaluation at 24 h after exposure	# Pretreated dexamethasone IP 5 mg/kg # Pretreated dexamethasone IT 0.1 or 0.5 mg/kg # Pretreated Sodium Cromoglycate IP 40 mg/kg for 1 h	# Dexamethasone IP: ↓ BALF cell count, PMN ↓ BALF and plasma histamine # Dexamethasone IT (0.5 mg/kg): ↓ BALF cell count, PMN ↓ BALF histamine ↔ plasma histamine # Sodium cromoglycate: ↓ BALF cell count, PMN ↓ BALF and plasma histamine	# Dexamethasone IP: ↓ thrombus formation # Dexamethasone IT (0.5 mg/kg): ↓ thrombus formation # Sodium cromoglycate: ↑ PFA100 closure time		Dexamethasone prevented PM-induced lung inflammation, histamine release, and thrombosis. Anti-inflammatory pretreatment also helped prevent PM-induced histamine release and reduced the prothrombotic state.	[68]
Male TO mice (HsdOla: TO) 30–35 g	Intratracheal instillation of DEP (SRM 2975) 15 µg/animal on day 0, 2, 4, 6 Evaluation at 48 h after the last exposure	Pretreated with Curcumin (200 µl) oral gavage for 1 h	BALF: ↓ PMN, macrophages ↓ TNF-α ↔ IL-6 Plasma: ↓ CRP ↓ TNF-α ↔ IL-6	↑ thrombotic occlusion time ↓ D-dimer ↓ PAI-1 ↔ VWF	↑ Platelet	Curcumin pretreatment prevented DEP-induced inflammation and promoted fibrinolytic activity, which diminished the prothrombotic state.	[66]
Male TO mice (HsdOla: TO) 30–35 g	Intratracheal instillation of DEP (SRM 2975) 30 µg/animal Evaluation at 4, and 18 h after exposure	Pretreated with anti-inflammatory agent: Thymoquinone IP 6 mg/kg for 1 and 24 h	BALF ↓ PMN, macrophages ↓ IL-6 ↓ total protein Plasma ↓ IL-6 ↑ superoxide dismutase	↑ thrombotic occlusion time	↓ WBC ↑ Platelets	Thymoquinone pretreatment significantly prevented DEP-induced inflammatory response, oxidative stress, and thrombosis.	[48]
Male TO mice	Intratracheal instillation of DEP 1 mg/kg Evaluation at 24 h after exposure	Emodin (antioxidant/anti-inflammation) IP 4 mg/kg twice, 1 h before, and 7 h after exposure	↓ TNF-α ↓ IL-1β ↑ superoxide dismutase ↓ glutathione reductase	↑ thrombotic occlusion time	↓ Hb, Hct, RBC ↓ WBC	Administration of antioxidants prevented DEP-induced inflammatory response, oxidative stress, and thrombotic complications.	[47]
DM type II patients (N = 30) Mean age 56.5 y	Acute exposure to ambient PM in Rochester, NY, USA Evaluated the PM level at 1, 12, 24, 48, 96 h	8 wk sequential therapy with # ASA 81 mg/d for 7 d # Fish oil 4 g/d for 28 d # Combined for 7 d		# ASA, and/or fish oil: ↔ TBXB2 ↔ ADP-, and collagen-induced platelet aggregation		ASA/fish oil blunted the effect of pollution on platelet function and TBXB2.	[78]

ADP: adenosine diphosphate, BAL: bronchoalveolar lavage, BALF: bronchoalveolar lavage fluid, BAT: brown adipose tissue, β_2_AR: adrenergic receptor beta-2, CAPs: concentrated ambient particles, CRP: C-reactive protein, d: days, DEP: diesel exhaust particles, DM: diabetes mellitus, h: hours, Hb: hemoglobin, Hct: hematocrit, IL-1β: interleukin-1β, IL-6: interleukin-6, IL-10: interleukin-10, IL-12: interleukin-12, IFN-γ: interferon-γ, IP: intraperitoneal injection, IT: intratracheally instillation, MCP-1: monocyte chemoattractant protein-1, mRNA: messenger ribonucleic acid, NE: norepinephrine, PAI-1: plasminogen activator inhibitor-1, PFA100: platelet function analyzer-100, PM: particulate matter, PM_10_: particulate matter in diameter <10 µm, PMN: polymorphonuclear cells, PT: prothrombin time, PTT: partial thromboplastin time, RBC: red blood cells, SRM: standard reference material, TAT: thrombin-antithrombin complexes, TBXB2: thromboxane B2, TF: tissue factor, TNF-α: tumor necrotic factor-α, TO mice: Tuck-Ordinary mice, VWF: von Willebrand factor, WBC: white blood cells, wk: weeks, WT mice: wide type mice, y: years.

## Data Availability

Not applicable.
